# A simple intervention for disorders of consciousness- is there a light at the end of the tunnel?

**DOI:** 10.3389/fneur.2022.824880

**Published:** 2022-07-22

**Authors:** Kudret Yelden, Leon M. James, Sophie Duport, Agnieszka Kempny, Simon F. Farmer, Alex P. Leff, E. Diane Playford

**Affiliations:** ^1^Neurological Rehabilitation, Royal Hospital for Neuro-Disability, London, United Kingdom; ^2^Department of Neuroscience, King's College Hospital, London, United Kingdom; ^3^UCL Queen Square Institute of Neurology, University College London, London, United Kingdom; ^4^Neurophysiology Department, Bupa Cromwell Hospital, London, United Kingdom; ^5^Research Department, Royal Hospital for Neuro-Disability, London, United Kingdom; ^6^National Hospital for Neurology and Neurosurgery, University College London Hospital, London, United Kingdom; ^7^Warwick Medical School, University of Warwick, Coventry, United Kingdom

**Keywords:** disorders of consciousness, sleep, circadian rhythm, event related potentials, melatonin, polysomnography, vegetative state, minimally conscious state (MCS)

## Abstract

Sleep is a physiological state necessary for memory processing, learning and brain plasticity. Patients with disorders of consciousness (DOC) show none or minimal sign of awareness of themselves or their environment but appear to have sleep-wake cycles. The aim of our study was to assess baseline circadian rhythms and sleep in patients with DOC; to optimize circadian rhythm using an intervention combining blue light, melatonin and caffeine, and to identify the impact of this intervention on brain function using event related potentials. We evaluated baseline circadian rhythms and sleep in 17 patients with DOC with 24-h polysomnography (PSG) and 4-hourly saliva melatonin measurements for 48 h. Ten of the 17 patients (5 female, age 30–71) were then treated for 5 weeks with melatonin each night and blue light and caffeine treatment in the mornings. Behavioral assessment of arousal and awareness [Coma recovery scale-revised (CRS-R)], 24-h polysomnography and 4-hourly saliva melatonin measurements, oddball mismatch negativity (MMN) and subject's own name (SON) experiments were performed twice at baseline and following intervention. Baseline sleep was abnormal in all patients. Cosinor analysis of saliva melatonin results revealed that averaged baseline % rhythmicity was low (*M*: 31%, Range: 13–66.4%, *SD*: 18.4). However, increase in % Melatonin Rhythm following intervention was statistically significant (*p* = 0.012). 7 patients showed improvement of CRS-R scores with intervention and this was statistically significant (*p* = 0.034). All the patients who had improvement of clinical scores also had statistically significant improvement of neurophysiological responses on MMN and SON experiments at group level (*p* = 0.001). Our study shows that sleep and circadian rhythms are severely deranged in DOC but optimization is possible with melatonin, caffeine and blue light treatment. Clinical and physiological parameters improved with this simple and inexpensive intervention. Optimization of sleep and circadian rhythms should be integrated into rehabilitation programs for people with DOC.

## Introduction

The number of patients who survive profound brain injuries is increasing. While some make good progress, others remain in a disorder of consciousness (DOC) which includes vegetative state/unresponsive wakefulness syndrome (VS/UWS) and minimally conscious state (MCS) (1, 2). Patients in VS/UWS do not show any sign of awareness of themselves or their environment while patients in MCS exhibit inconsistent but clearly discernible behavioral signs of awareness such as visual tracking, simple command following and gestural yes/no responses.

Patients in DOC usually have suffered widespread damage to their cerebral hemispheres and connections which leads to impairment of consciousness, but have relative sparing of hypothalamic and brain stem structures which maintain arousal and autonomic functions (3). They exhibit periods of eye closure and eye opening resembling sleep and wake cycles; however, their sleep architecture may not be normal. Recent studies have demonstrated a lack of typical neurophysiological sleep patterns in DOC (4–6). Furthermore, it has been shown that the presence of sleep spindles, slow wave sleep and rapid eye movement (REM) sleep correlate positively with clinical scores of awareness (7–9). Structured sleep is associated with positive clinical outcomes (10).

Regulation of sleep is complex and depends on close interactions between endogenous and exogenous factors. The suprachiasmatic nucleus is the main endogenous pacemaker that orchestrates synchrony among the circadian oscillators of peripheral organs such as the liver, lungs, heart, and kidneys, as well as limbic, cerebral, and cerebellum brain (11). Exogenous components are driven by the individual's lifestyle and environmental factors (12, 13). Abnormality of one or more of these factors may lead to dysregulation of circadian rhythms. Deranged circadian rhythmicity leads to sleep abnormalities which in turn, negatively affect brain functions. Growing evidence suggests that REM sleep may play a vital role in learning and memory formation (14–18), and sleep deprivation impairs learning, memory, and other cognitive abilities (19). Hence, it is possible that impaired circadian rhythmicity and abnormal sleep may worsen awareness and other cognitive functions in patients in DOC.

Patients with a DOC are often looked after in an institutional setting where environmental factors are not conducive to maintaining circadian rhythmicity (20). They may be prescribed central acting medications that influence sleep-wake patterns and are fed *via* feeding tubes continuously or at night which is likely to impact negatively on the relationship between the suprachiasmatic nucleus and peripheral circadian clocks located in the liver, pancreas and intestine (21, 22).

Therefore, it is important to make attempts to improve sleep-wake patterns and circadian rhythms of patients with DOC. However, a recent systematic review found that only 7 papers which focused on pharmacological and non-pharmacological sleep treatments in pediatric and adult patients with DOC, including the sleep disordered breathing (23). Majority of these papers were reports of single cases. The treatments used in these studies were modafinil (24, 25), intrathecal baclofen (26), positive airway pressure (27), bright light stimulation (28), and central thalamic deep brain stimulation (29, 30).

Given that environmental factors, as well as the brain injury itself, can affect circadian rhythms in DOC patients, we decided to intervene in three ways, using melatonin, light and caffeine. Melatonin has been shown to be beneficial in patients with sleep disorders and with Alzheimer's Disease (31, 32) and a case study indicates that it was given to help with deranged sleep-wake cycle of a 15-year old patient with DOC (24). Blume et al. piloted bright light stimulation for 1 week on eight patients with DOC and found evidence of improvement of circadian rhythm as evidenced by shift of body temperature maximum to afternoon hours from early morning or evening hours, as well as increase of CRS-R scores in 3 patients (28). Caffeine is a weak psycho-stimulant and can counteract deteriorations in task performance related to sleep deprivation (24). Subjects who suffered from jet lag achieved resynchronization of their circadian rhythm earlier than placebo group subjects when given caffeine alongside melatonin (25, 26).

As well as using a standard behavioral assessment to measure the effect of our tripartite intervention, we also wished to see if there were any significant effects on the patients' brain function. Event related potential (ERP) studies have been used to detect the presence of higher-level cerebral functions in patients with DOC (28–34). A variety of paradigms have been used and we chose two of the commonest, an auditory Mismatch Negativity (MMN) paradigm using simple tones, and a subject's own name paradigm. Both produce electrical signatures in normal subjects that require multifocal cortical processing and integration. Both ERP-inducing paradigms have some power at predicting recovery (35–37).

The aim of our study was to assess baseline circadian rhythms and sleep in patients with DOC; and, if found to be abnormal, to optimize it using the three interventions mentioned above. Our three outcomes were clinical (CRS-R), circadian (polysomnography and melatonin levels) and electrophysiological (MMN and own name ERPs).

## Methods

### Setting and patients

This study was conducted in the rehabilitation and long-term care units of a specialized center in London. The rehabilitation unit admits patients with DOC for a period of 3–6 months to provide specialist diagnostic assessments and rehabilitation. The long-term care units provide specialist nursing care for people with profound brain injuries.

A total of 17 patients (7 female, aged 30–73) with DOC (5 VS/UWS, 12 MCS) were included in the baseline studies which aimed to investigate the circadian rhythm and sleep pattern/structures. Aetiological causes for the brain injury included anoxic brain injury (*n* = 8), trauma (*n* = 3), cerebrovascular accident (CVA) (*n* = 3), mixed pathology (*n* = 2) and vasculitis (*n* = 1). Five of the 17 patients were discharged upon completion of their assessments and rehabilitation therefore, not included in the interventional study. Of the 12 remaining patients, one died during the intervention due to an unrelated medical condition (patient 12) and another was excluded due to frequent involuntary movements which affected electroencephalogram (EEG) quality (patient 16), leaving 10 patients who completed the intervention part of the study. The 10 patients who took part in the interventional study were not receiving any other rehabilitation or treatment which could lead to improvement of their consciousness levels. They all had post-acute rehabilitation and consciousness assessments at the same institution within the first 12 months of the brain injury using Sensory Modality Assessment Rehabilitation Technique (SMART) (33, 34) which is a standardized, very detailed and structured assessment tool. The patients were then transferred to the long-term care wards where they were monitored using regular assessments using standardized assessment tools including Wessex Head Injury Matrix (WHIM) (35), CRS-R (36) or repeat SMART assessments throughout their stay. None of the patients had documented improvement of their consciousness levels within the preceding 12 months of our study. Detailed demographic and clinical information which include the etiology and time since the brain injury are summarized in Table 1.

**Table 1:** Patient information.

**Patient ID/** **age/gender**	**Etiology**	**Diagnosis**	**BL CRS-R Score**	**Experimental Condition**	**Time since BI**	**Setting**	**Feeding regime**	**Medications**	**CT/MRI**	**BAEP**	**EEG BL**
Patient-1 71/Female	CVA	MCS	12	BL and Tx	5 years	LTC	Daytime	Co-careldopa, levetiracetam, lansoprazole	Extensive white matter low attenuation, atrophy, R frontal bleed, L occipital infarct, VP shunt	Normal	Polymorphic delta activity at 2–2.5 Hz at 20–30 μV, small amounts of beta
Patient-2 58/Male	Anoxia	MCS	8	BL and Tx	5 Years	LTC	Daytime	Amiodarone, frusemide, ramipril, valproate, levetiracetam, diltiazem, clonazepam, levothyroxine	Widespread ischaemic changes, loss of gray-white matter differentiation in cortex and basal ganglia	R absent, L present	Polymorphic delta activity at 1–2.5 Hz at 10–20 μV, some beta, sharp waves/ spikes
Patient-3 36/ Male	Anoxia	VS	4	BL and Tx	2 years	LTC	Daytime	Lansoprazole, metformin, aspirin, simvastatin, bisoprolol, clonazepam, dantrolene, zopiclone	Widespread changes	Normal	Frequent spike wave discharges on low amplitude featureless background
Patient-4 52/Male	CVA	MCS	10	BL and Tx	4 years	LTC	16:00–04:00	Baclofen, dantrolene, gabapentin, valproate, lansoprazole	Right temporal AVM, intracerebral and subdural hemorrhage	R absent, L present	Asymmetric EEG, Right: low amplitude featureless, Left: theta and delta
Patient-5 30/Female	TBI	MCS	13	BL and Tx	5 years	LTC	16:00–02:00	Lansoprazole, hyoscine, baclofen, gabapentin, citalopram	Multiple widespread petechial hemorrhage	Normal	EEG was dominated by delta activity at 1.5–2.0 Hz, up to 50 μV, ripples of theta
Patient-6 43/Female	CVA	MCS	16	BL and Tx	3 years	LTC	18:00–02:00	Amlodipine, doxazocin, levetiracetam, oxybutynin, simvastatin	Left temporo-parietal stroke & hemorrhage	R delayed, L Normal	Asymmetric EEG, dominant delta and theta activity on the right
Patient-7 73/Male	CVA	MCS	12	BL and Tx	8 years	LTC	16:00–02:00	Bisoprolol, clopidogrel, gabapentin, warfarin	Bilateral ganglia infarcts, and infarction in the left occipital lobe	Normal	Diffuse theta, occasional delta 1–2.5 Hz
Patient-8 40/Female	TBI	MCS	11	BL and Tx	3 years	LTC	Daytime	Baclofen, hyoscine, lansoprazole, modafinil	Multiple widespread petechial hemorrhage	Normal	Diffuse theta, occasional delta
Patient-9 68/Male	Vasculitis	VS	6	BL and Tx	2 years	LTC	16:00–04:00	Bisoprolol, pregabalin	Widespread necrotising leuco-encephalopathy	R absent, L present	Diffuse delta 1–2,5 Hz, occasional theta
Patient-10 66/Female	Anoxia	MCS	11	BL and Tx	6 years	LTC	Daytime, bolus	Clobazam, diazepam levetiracetam, mebeverine, mirtazapine, omeprazole, paracetamol, ramipril, zopiclone, amlodipine, atorvastatin	extensive diffuse signal change in the left temporal lobe, the left occipital and parietal cortices consistent with ischaemic damage	Normal	Copious amounts of beta activity seen at 15–17 Hz
Patient-11 59/Female	Anoxia	VS	5	BL only	6 months	Rehab.	10:00–24:00	Clonazepam, baclofen, hyoscine patch, dantrolene	Diffuse hyperintense signal at bilateral periventricular and deep white matters, basal ganglia and occipital gyri.	N/A	Poorly responsive EEG, frequent spikes, diffuse irregular delta activity at 2 Hz
Patient-12 59/Female	Anoxia	MCS	13	BL only	8 years	LTC	Daytime	Lansoprazole, tizanidine, laxatives	Diffuse cortical and deep gray matter high signal abnormalities with swelling. Global cerebral infarction and hypoxia/ischemia	R absent, L normal	Diffuse theta, occasional delta 1–2.5 Hz
Patient-13 49/Male	Anoxia	VS	7	BL only	9 months	Rehab.	Daytime	Baclofen, warfarin, dantrolene, levetiracetam, clonazepam	Global hypoxic brain injury	N/A	background theta activity at 5–6 Hz, Occasional delta at 1.0–2.0 Hz, Widespread beta at 18–20 Hz was seen.
Patient-14 55/Male	CVA (haem)	MCS	10	BL only	9 months	Rehab.	16:00–04:00	Folic acid, lansoprazole, levetiracetam, bisoprolol, thiamine, amlodipine, ramipril, baclofen	Left intracerebral hemorrhage, cerebral atrophy, ischaemic changes	R delayed, L absent	Diffuse delta activity at 1.5–2.0 Hz, Theta activity at 5–6 Hz over the central regions
Patient-15 60/Male	Anoxia	MCS	11	BL only	6 months	Rehab.	24:00–06:00 + x2 bolus	Aspirin, levetiracetam, pregabalin, simvastatin, alendronic acid	Global hypoxic brain injury	N/A	Poorly responsive EEG, central-posterior theta, Widespread/ patchy beta, posterior delta
Patient-16 38/Male	Anoxia	MCS	15	BL only	4 years	LTC	16:00–02:00	Baclofen, clonazepam, gabapentin, lansoprazole, levetiracetam, lorazepam, trihexyphenidyl	CT: no specific pathology	Inconclusive (difficult study)	N/A
Patient-17 53/Male	TBI	MCS	13	BL Only	7 months	Rehab.	16:00–04:00	Baclofen	Extensive oedema, large left parietal haematoma, contra-coup traumatic hemorrhage, SAH	Normal	Beta activity at 15–16 Hz over both hemispheres. Occasional theta and alpha

*BL, Baseline; BI, Brain injury; LTC, Long-term care; BAEP, Brainstem auditory evoked potentials; CVA, Cerebrovascular accident; Tx, Treatment; TBI, Traumatic brain injury; SAH, Subarachnoid hemorrhage (Patients 1–10 received intervention)*.

Ethics approval was obtained from London Queen Square Research Ethics Committee (Ref: 11/LO/1233), patients' relatives were provided with a study information sheet, acted as consultees and signed a record of consultation form prior to the patient's enrolment in the study.

### Study protocol

**Figure 1:**
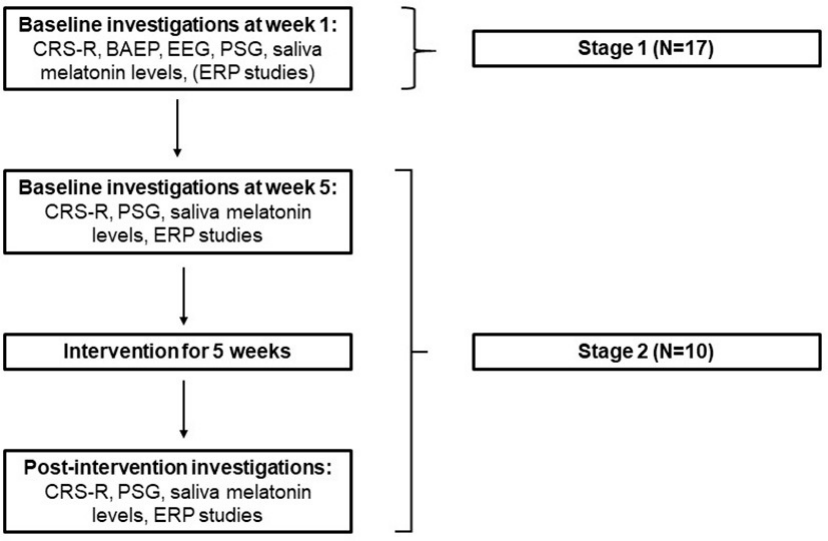
study design (PSG, Polysomnography; ERP, Event related potentials; CRS-R, Coma recovery scale- revised; BAEP, Brain stem auditory evoked potentials).

This study was performed in 2 stages, as outlined in Figure 1.

### Demographic data collection and bedside clinical assessment

All patients' electronic and paper based clinical files were reviewed. Clinical and demographic information were recorded on Excel (Microsoft Office 365, 2016).

The Coma Recovery Scale-Revised (CRS-R) is a standardized assessment tool that is used to aid diagnose and monitor DOC. It consists of 23 items that comprise six subscales addressing auditory, visual, motor, communication and arousal functions (36). CRS-R assessments were performed by two trained experts (KY and AK) at the beginning of each investigation week. The CRS-R assessments were undertaken in a quiet, well lit room while patients were in the sitting position, on the same day of the ERP experiments. CRS-R scoring sheets were completed upon consensus of both clinicians on patients' responses during the assessment. It was decided that if there was a disagreement on scoring individual trials of CRS-R subscales, the assessors would opt for the lower assessment. In our study the assessors did not have any disagreement which could change the final subscale scoring.

### Electrophysiological tests data collection and analysis

#### Electroencephalogram

The instrumentation used consisted of a Video-EEG monitoring system (XLTEK, Optima Medical, Guilford, UK) utilizing a 50-channel amplifier with a desktop computer integrated digital signal processor board utilizing Neuroworks Acquisition Software installed onto a windows XP operating system (XLTEK, Optima Medical, Guilford, Surrey, UK).

Baseline EEG investigations for 30 min were performed in order to assess electro-physiological features of brain and to exclude presence of non-clinical status epilepticus.

The EEG was recorded through the application of single use Ag/AgCl electrodes (Unimed Electrodes Ltd, UK) applied to the scalp in accordance with the 10–20 system of electrode placement. EEG electrodes applied were Fp2, Fp1, F8, F7, F4, F3, C4, C3, T4, T3, P4, P3, O2, O1, Fz, Cz, Pz, A2 and A1.

All electrode impedances were kept to below 5kΩ. Acquisition of the EEG signals was conducted with an EEG system which incorporated a 50-channel amplifier with a desktop PC integrated DSP board utilizing Neuroworks Acquisition Software installed onto a windows XP operating system (XLTEK, Optima Medical, Guilford, Surrey, UK). The EEG signals were online acquired with the common average reference montage, band pass filtered between 0.5–70 Hz, and sampled at a rate of 256 Hz without mains suppression.

#### 24 H polysomnography

Patients continued with PSG after 30 min of electroencephalography (EEG) with the same electrode scalp placement.

For the PSG study, the Trex HD ambulatory EEG headbox (XLTEK, Optima Medical, Guilford, UK) was used which incorporated polygraphic inputs for the measurement of additional physiologic parameters.

Measurement of electrooculography (EOG) was achieved by applying electrodes over the right (1 cm above) and left (1 cm below) outer canthi. Other physiologic parameters measured included submental surface electromyography (EMG), surface EMG taken from the right and left tibialis anterior and Lead I electrocardiography (ECG).

Respiratory effort was measured using Inductive Plethysmography Effort Sensors (Sleep Sense SLP inc, USA) with the effort belt positioned at the level of the xiphoid process. We were not able to measure the flow rate and sleep oximetry as part of the PSG studies.

The sampling rate for acquisition of all signals was 256 Hz. Filter settings for the on-line acquisition of the EEG and ECG signals was 0.5–30 Hz, sensitivity was 70 uV/cm. The filter settings employed for EMG signals was 10–100 Hz. Sensitivity settings for submental EMG was 20 and 100 uV/cm for EMG taken from tibialis anterior. Respiratory effort filter settings and sensitivity settings were 0.5–30 Hz and 0.5 mV, respectively.

During the PSG recording nursing staff were asked to fill in activity log where any event which may have attributed to awakenings and/ or artifacts were recorded.

The baseline EEG's recorded in the DOC patients were grossly abnormal. On preliminary review of the PSG EEG data, the features of the EEG which delineate the onset of sleep were sometimes absent or nebulous. Once the broad EEG patterns were identified, detailed visual inspection and micro-structure assessment of sleep recording were essential for sleep stage scoring by two experienced neurophysiologists who were both blinded to study stages that the patients were at, and to each other. The sleep EEG recordings were examined with the view of assessing the presence or absence of various sleep stages by identifying the micro-structural electrical signatures of different sleep stages such as sleep spindles, K complexes, conjugate EOG movements.

When visualizing the EEG and scoring the epochs, additional markers of wakefulness were adopted. These were taken as patterns of EMG artifact superimposed on EEG derivations and EOG activities, which were indicative of wakefulness in this cohort. The identification of the dissolution of the additional markers of wakefulness was taken to signify the sleep onset period, especially in those subjects where the differentiation in terms of EEG activities was nebulous. With some of the DOC subjects, the differentiation between awake and sleep EEG was clear. Amelioration of EMG artifacts, and changes in the EOG (eye blink deflections evolving into slow lateralised eye movements) could also be aligned with changes in the EEG denoting the onset of sleep. From this point, the epochs were scored as stage 1, until the sleep spindles were seen and other EEG changes, which demonstrated the progressive evolution of sleep EEG record. Total sleep time over 24 h and between 8 pm and 8 am, sleep efficiency, time spent in identified sleep stages (stages 1, 2, 3, REM) were recorded.

#### Brainstem auditory evoked potentials

Auditory brain stem evoked potentials testing was performed in order to ascertain ability of hearing through at least one ear.

BAEP were measured from each subject using Dantec KeyPoint 4 channel amplifier (Natus Medical, Inc., USA). Stimuli were delivered monoaural stimulation of each ear *via* headphones. The stimulus frequency was 10 Hz using alternating clicks (condensation/rarefaction) and intensity was 80 dB. The click stimulation was a square wave pulse of duration 100 μs.

Peak latency measurements of I, III and V, amplitude measurements of waves I and V, and inter-peak interval calculations of I-III and III-V were performed.

#### Event related potentials

All experiments were performed while patients were sitting in their own wheelchair in a quiet room. EEG activity was recorded using 64-channel EEG amplifier (ANT-Neuro, Netherlands) and shielded waveguard EEG cap. To optimize signal quality, the impedance at electrodes were kept below 5 kΩ. Sampling rate was 1024 Hz and two of the electrodes were used to record EOG.

Two experiments were used to obtain ERP components were MMN and SON paradigms. Patients were provided with 5–10 min rest time between the two experiments. The experimental order was not randomized across subjects or the three timepoints (e.g., SON first then MMN) but stimuli were randomized within each session.

During the frequency oddball paradigm, the presented stimuli were 1000 pure tones of 1,000 Hz (80% standard) and 1,200 Hz (20% deviant), with an intensity of 70 dB and duration of 60 ms (rise time 10 ms), delivered binaurally through headphones. Inter-stimulus interval was 500 ms.

In the subject's own name paradigm, three different types of stimuli were used: (1) subject's own name; (2) four other peoples' names; and, (3) four time-reversed other names. Other names were chosen from the pool of names which were not significant to the patients. Other names were time reversed to create unintelligible stimuli which preserve their acoustic complexity and voice identity. The names were recorded by a native English speaker male person unknown to them and edited for quality and length (range 400–800 ms), time-reversed and amplified using Praat software version 5.3.23 (38). The stimuli were delivered binaurally *via* headphones. The stimuli were presented in a random order and the same stimulus could not be repeated successively. The number of stimuli for each of the three conditions were: 110 own names, 471 other names and 419 reversed names. Inter-stimulus interval was 1,200 ms. The duration of the experiment was ~20 min.

### Measures of circadian rhythm and sleep

#### Melatonin as a marker of circadian rhythmicity

**Figure 2:**
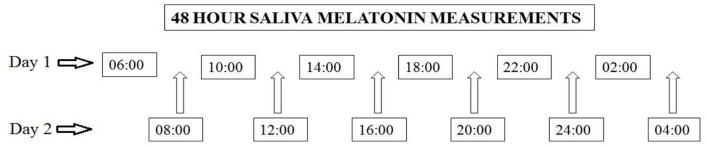
Saliva melatonin collection protocol.

Saliva melatonin samples were collected using Salimetrics pediatric oral swabs (Salimetrics, USA) over 48 h during baseline-1 and baseline-2 investigations. In a 48-h period samples were collected every 4 h with a shift of 2 h on collection times in the second half (see Figure 2).

During the intervention, 8 am and 8 pm samples were collected to confirm presence of high and low saliva melatonin levels. 24-h saliva collection were performed 3 days after cessation of intervention to find out whether the circadian rhythm of melatonin secretion was entrained and once again 2–3 weeks after to see if this was maintained or not.

After collection, the samples were frozen and later shipped for measurement of the melatonin level using radioimmunoassay analysis at the laboratories of Stockgrand Ltd. (University of Guilford, Surrey, UK). The assay characteristics were as follows: limit of detection = 0.9 pg/ml, quality controls low = 3.6 pg/ml +/- 0.4 CV = 10.7%; medium = 26.1 pg/ml +/- 2.3 CV = 8.9%; High = 54.6 pg/ml +/- 4.9 CV = 9.0%.

### Circadian intervention

The intervention consisted of treatment with melatonin, caffeine and light for 5 weeks. The intervention was not scheduled at a particular circadian phase of the individual patients but timed to mimic normal physiological states of melatonin rise and suppression.

#### Melatonin

In order to synchronize environmental cues, light-dark cycle of their environment and sleep-wake cycle, melatonin administration time was set as 6 pm in the evenings. Liquid 3 mg Melatonin (1 mg/ml) was given *via* percutaneous enteral gastrostomy (PEG) tube at 6 pm each night by nursing staff during their medication rounds.

#### Light treatment

Light treatment was given using Philips Energy Light lamps (HF 3,308) which mimic natural daylight by giving up to 10,000 lux at 20 cm and 2,500 lux at 60 cm distance. Prior to use in the study, the lamps were tested in the laboratories of Health Protection Agency and results were published (37). Lamps were placed at average distance from patients' eye level of 60 cm. The light treatment was given between the hours of 08:00 and to 09:00 every day, for 1 h.

#### Caffeine

We used ProPlus Caffeine tablets (Bayer plc.) The starting dose of the caffeine was 50 mg. This was increased by 50 mg every 3 days to treatment dose of 100 mg twice a day (at 08:00 and 12:00 h) which was the dose given for the rest of the intervention period. Caffeine tablets were crushed and dissolved according to usual nursing practice of giving tablets *via* feeding tube and after administration the tubes were flushed with water to avoid tube blockage.

### Statistical analyses

Descriptive statistical tests, means comparisons (*t*-test and Wilcoxon signed ranks test), ANOVA tests and reliability analyses on the clinical data were performed using SPSS software (IBM Corp. 2016. IBM SPSS Statistics for Windows, Version 24.0. Armonk, NY).

Raw melatonin data were normalized to the mean and plotted as a function of time, to explore whether an obvious rhythmicity could be recognized or not; to make macroscopic/qualitative analysis of noise and, to see if there was cycle-to-cycle variability or not. Examination of maximum (peak) melatonin values were made. Normalized melatonin values were further analyzed using single cosinor analysis software which was provided by Stockgrand UK laboratories. The software is an Excel based program which calculates rhythmicity expressed as a percentage from the values of circadian rhythm markers. Percentage rhythm is the measure of the relative strength of a rhythm and is calculated by making direct comparison between the variability of the data points about the fitted curve (39).

For the patients who were included in the interventional study General Linear Model (GLM) ANOVA tests were performed to see if the data differed at three time points (baseline 1, baseline 2 and post-intervention) and pairwise comparisons were made for CRS-R total scores, PSG parameters and melatonin % rhythm. Where the baseline 1 and 2 data were not significantly different at 0.05 level (CI = 95%), the baseline data were collapsed together and means comparisons were made against post-intervention data.

The PSGs were reported by two neurophysiologists who were blinded to each other, the PSG data obtained two-way mixed intra-class correlations (raters are chosen and subjects are random) were performed to assess inter-rater reliability. If the absolute agreement was at and above acceptable level (>0.7, CI = 95%), the data from both raters were collapsed together and *t*-test comparisons were made between pre-intervention and post-intervention results.

ERP data pre-processing and analysis were performed offline using Statistical Parametric Mapping (SPM12, Institute of Neurology, UCL) software (40, 41). Following data pre-processing, 3D images were created for each trial, containing smoothed recorded potentials in scalp space (two dimensions, X and Y) and over peri-stimulus time (z dimension). *T*-test comparisons were made between averaged baseline conditions and post-intervention for the patients who had improvement of CRS-R scores to see if the improvements were also reflected in electrophysiological tests. We searched across the whole of scalp space but, limited the analysis to a fixed time window of interest 150-500 ms post-stimulus (by applying a small volume correction). After this mask was applied to the SPM, we report voxels that survived a peak level value of *p* < 0.001 uncorrected.

Contrasts for each subject were created at the first level that identified the ERP of interest, creating a single statistical map for each subject, for each time point (see Table 2). These were then taken up to a second level design matrix (paired *t*-test) to examine statistical differences between the responses before and after the intervention using appropriate contrasts.

**Table 2:** Contrasts applied for oddball and subject's own name experiments at the 1st level design matrices.

**ERP Test**	**Time points that the contrasts applied**	**1st level individual design matrix for 3 time-points**
Oddball	Baseline experiments	−1 1−1 1 0 0
	Post-intervention experiments	0 0 0 0−1 1
SON	Baseline experiments (SON> OTHER)	1−1 0 1−1 0 0 0 0
	Post-intervention experiments (SON> OTHER)	0 0 0 0 0 0 1−1 0

*SON, Subject's own name. NB: there were two baseline time points and one post-intervention time point*.

In order to extract *post-hoc* MMN values after the intervention, the individual subjects' 1st level design matrix were interrogated and the peak MMN values closest to the group peak and extracted the beta values (in μV) were searched.

## Results

### Baseline investigations results

#### Brainstem auditory evoked potentials

We were able to obtain BAEP testing in 13 patients (one study difficult/inconclusive, three not done due to missed appointments). While seven out of 13 patients had completely normal results, the remaining six patients had some abnormality in their BAEP results. None of the patients had hearing impairment affecting both sides. All 10 patients who proceeded to interventional study were tested and three of them (patient 2, 4, and 9) had absent wave 1 on the right side but normal BAEP responses on the left.

#### Electroencephalogram

Baseline EEG features have been summarized in Table 1.

#### Melatonin as a marker of baseline circadian rhythmicity

**Figure 3:**
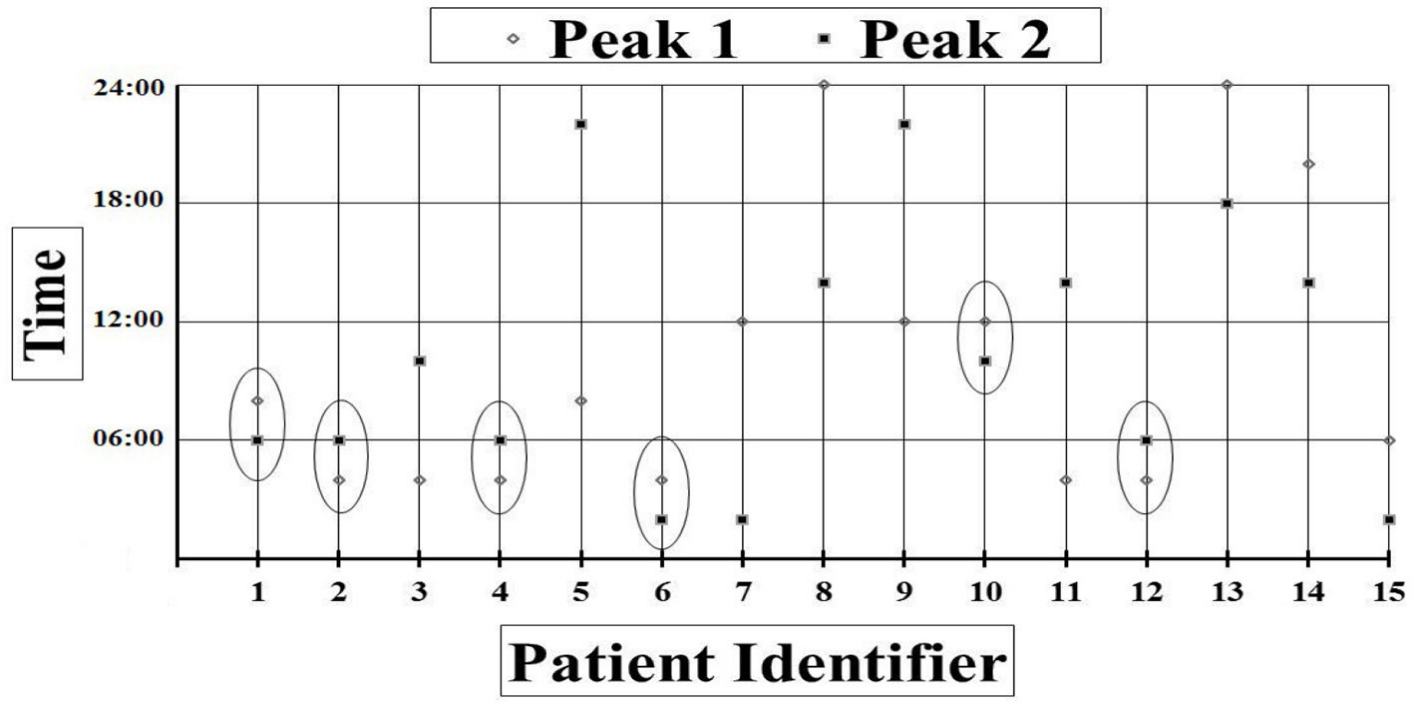
Peak times in each consecutive 24-h period for each patient. Peak times occurring within the same 2-h window are circled.

Forty-eight-h saliva melatonin results were available for 15 patients. Out of the 180 samples collected within 48 h 158 samples yielded results. Review of the melatonin plots showed that 6 (1 VS, 5 MCS) out of 15 patients had melatonin peak times occurring within the same 2-h window on consecutive 24-h periods indicating that these patients may have a degree of preserved circadian rhythmicity. However, timings of the melatonin peaks were during daylight hours for two of those patients (around 7 and 11 am) (see Figure 3).

Daytime (8 to 8 pm) melatonin mean was 84 pg/ml (*SD* = 29.4), and night (8 pm−8 am) melatonin mean was 107 pg/ml *(SD* = 27.8). Means comparisons of day and night normalized melatonin values did not reach statistically significant values (*p* = 0.07).

Cosinor analysis of saliva melatonin results revealed that averaged baseline % rhythmicity was low (*M*: 30.87%, Range: 10–66.4%, *SD*: 16.5). % Rhythmicity was higher in the patients who were fed during the day than in the patients who were fed at night but the difference within the two groups was not statistically significant (*p* = 0.22).

#### Polysomnographic assessments

The PSG reports were available for 15 patients. Respiratory effort measurements did not indicate presence of any obvious central or obstructive apnoeic episodes. Total recording time for the PSGs varied between 1,247 and 1,855 min, therefore, total sleep time and sleep efficiency values were normalized to 24-h period. Total sleep time was reduced (Range: 34–518 min, *M*: 246 min, *SD*: 146.4).

None of the baseline PSGs contained stage 3 sleep. Only 4 out of 15 patients showed any evidence of short periods of REM sleep. The patients who had evidence of REM sleep were in MCS.

### Interventional study results

Following 5-weeks of intervention there were statistically significant improvements in: CRS-R scores, melatonin rhythmicity and event-related measures of pre-attentive sensory processing.

#### Clinical outcomes

Seven of the 10 patients improved their CRS-R scores following intervention. None of the patients had a clear change of diagnosis from VS to MCS or MCS to “emergence from MCS.” A Wilcoxon signed ranks test revealed this to be a statistically significant effect; both between CRS-R baseline averaged data and post-intervention (*p* = 0.034) and, between baseline-2 and post-intervention which represents the change between the two closest time points before and after the intervention (*p* = 0.027) (Table 3).

**Table 3:** Mean and interquartile range (IQR) values for CRS-R (*n* = 10).

	**Mean**	**IQR**
Baseline-1	10.6	4
Baseline-2	10.2	4
Baseline-averaged	10.4	4.5
Post-intervention	11.8	6

#### Melatonin

GLM pairwise comparisons between baseline 1 and baseline 2 conditions were not significant for melatonin rhythms. Hence, the data from baseline conditions and post-intervention conditions were collapsed together and statistics were performed.

**Figure 4:**
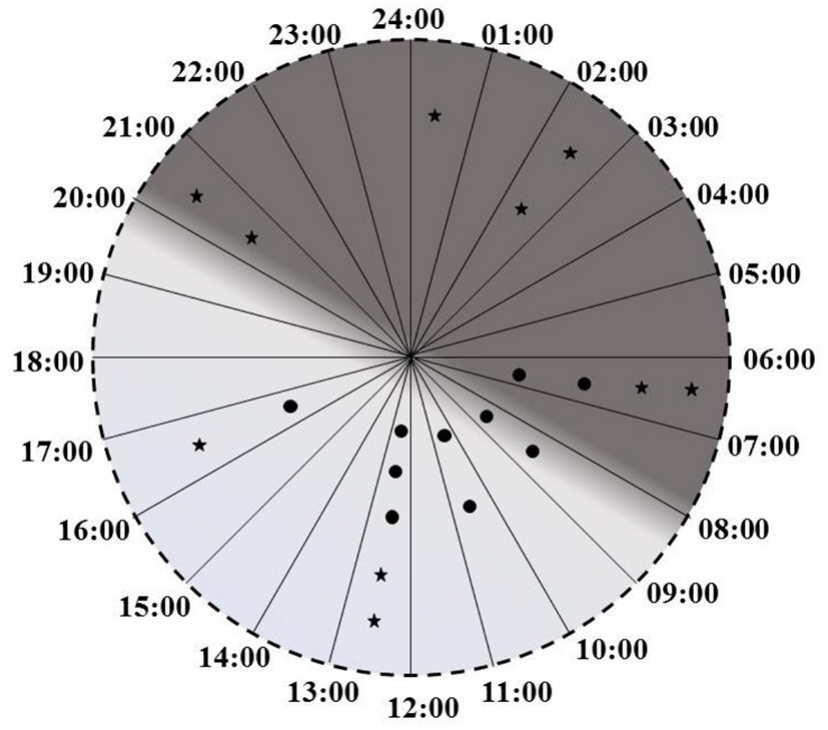
Melatonin peak times before the interventions (circles) and after the intervention (stars).

Increase in % Melatonin Rhythm following intervention was statistically significant *t*_(9)_ = 3.13, *p* = 0.012. Improvement in % melatonin rhythm was maintained even 3 weeks after cessation of intervention. A paired *t*-test on data collected at 3-days and 3-weeks post intervention was not significant [*t*_(9)_ = 0.15, *p* = 0.89]. Plotting of peak times revealed that while prior to intervention only 2 patients had melatonin peaks in dark, after the intervention 7 patients had their melatonin peaks in dark (Figure 4).

#### Polysomnography

Pairwise comparisons between baseline 1 and baseline 2 conditions were not significant for any of the PSG parameters. As the PSG studies were independently reported by two different neuro-physiologists who were also blinded to patient's experimental conditions and to each other, intra-class correlations were performed to see if there was an absolute agreement between them. It was found that there was excellent agreement for total sleep time, time spent in sleep at night, sleep efficiency and REM stage scorings. Therefore, the two baseline measures were collapsed together and compared in a paired *t*-test with the post-intervention data. Means comparisons were made to see if the improvements observed were statistically significant. Although improvement of all 4 parameters (total sleep time, time-spent in sleep at night, sleep efficiency and REM) were observed the improvement following the intervention failed to reach statistically significant levels.

#### Event-related potentials

At the group level, the patients who had improvement of clinical scores (7/10) also had statistically significant improvement of neurophysiological responses on MMN and SON experiments (*p* = 0.001).

**Figure 5:**
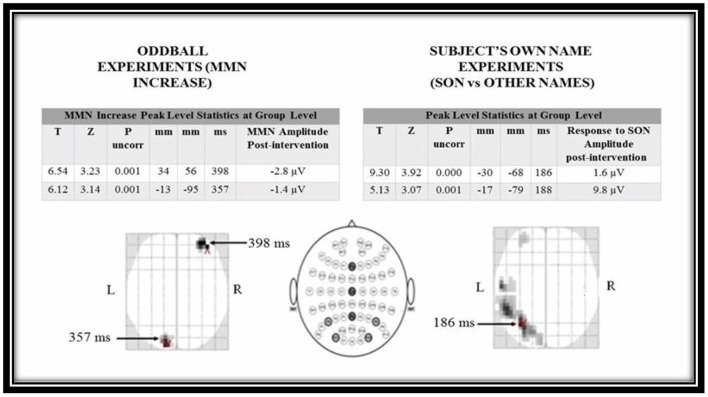
Statistical parametric mapping *t*-test results for oddball and subject's own name experiments for pre-intervention and post-intervention conditions. MMN, mismatch negativity; SON, Subject's own name.

At the group level we identified two peaks where the MMN increased after therapy. These were in the right frontal region and left occipital region. In the SON experiment we identified two peaks response to stimuli amplitude increased with therapy. These were both located at the left parietal region (see Figure 5).

None of the three non-responders had any significant ERP results that overlap in space or time with the group effect seen in the responders in the MMN experiment. For the SON experiment, one patient had no suprathreshold clusters. The other two patients had suprathreshold clusters in the left hemisphere however, they were considerably later on in time.

## Discussion

In this study, we were able to improve circadian rhythm in patients with DOC by using melatonin and bright light treatment as chronobiotic agents and caffeine as an adjuvant to inhibit melatonin production (and by blocking adenosine receptors as a neurostimulant). This was associated with a significant improvement on the CRS-R. A sub-analysis of the brain imaging data from those who responded clinically showed a commensurate increase in both of the neurophysiological measures of cortical function (MMN and SON). To our knowledge, this is the first study of an intervention focussed on circadian rhythm which is associated with a change in arousal and awareness using both behavioral (CRS-R) and neurophysiological measures.

These findings contrast those of Guaraldi's study (42) in which 6 traumatic VS patients failed to supress melatonin levels when exposed to blue light. This lack of response may well have been due to damage to the hypothalamic and midbrain axes in these more severely affected patients. The differences between their study and ours may be explained in a number of ways. Their cohort may have had more severe brain injury, they used blue light for 2 h for one night to try to suppress melatonin levels. In contrast we treated patients for 5 weeks, and the changes will have been supported by the ward routine. In addition, following a previous study which examined the light and noise levels on wards at night (20) we had already undertaken strenuous efforts to ensure a dark, quiet, comfortable ward at night.

More importantly, in our view, the combination treatment with melatonin, blue light and caffeine might have enhanced the positive responses in our outcome measures. There are intricate and complex interactions between melatonin, light and caffeine (43, 44). Adenosine acts on sleep-inhibiting and sleep-initiating regions of the brain. It encodes sleep history and this signal modulates circadian entrainment by light (45). Adenosine levels build up during the day and peak usually 12–16 h of being awake in people with normal circadian rhythm. This creates the sleep pressure (desire to sleep). Caffeine binds adenosine receptors and inactivates them (46). Therefore, despite building up of adenosine levels, brain does not get the desire to sleep. This continues as long as there are enough caffeine molecules binding to the adenosine receptors. Once the caffeine is metabolized, the adenosine freely binds its own receptors which creates high sleep pressure. By giving caffeine as part of the intervention, we might have facilitated increased alertness/less urge to sleep during daytime and increased sleep pressure at night. It is possible that this might consequently have led to production of more consolidated sleep.

Our baseline PSG studies showed that sleep-wake patterns of our patients (*n* = 15) were grossly abnormal. Previous studies (5, 7) have found that REM sleep is more common in MCS than in VS and this pattern was re-iterated in our study as all four patients who had REM sleep were in MCS. However, the amount of REM was much less than that accepted as normal which may impact on memory consolidation and learning.

We were not able to measure the flow rate and sleep oximetry as part of the PSG studies. While review of the respiratory effort using plethysmography sensors did not show any obvious apnoeic episodes, we cannot fully exclude central or obstructive sleep apnoea episodes due to lack of flow rate and sleep oximetry during our PSG studies. We recommend that full respiratory parameters are included in future PSG studies in this patient group, as there is a growing evidence that sleep disordered breathing is common in brain injury patients and negatively impacts on recovery and neural repair (47).

Our findings of an association between improved circadian rhythm and arousal has been observed in other settings. It has been suggested that the presence or absence of circadian rhythms can be used as a marker of prognosis as they reflect hypothalamic and midbrain functions (48). DOC patients are likely to have abnormal circadian rhythms, which may differ between VS and MCS patients. Blume et al. found that in 20 DOC (13VS, 7 MCS) patients the maintenance of circadian rhythm was associated with a richer behavioral repertoire (49), and Gobert observed that return of circadian rhythm was associated with a better prognosis indicating transition from VS to MCS (50).

One of the challenges for clinicians is providing an accurate prognosis for people with a profound brain injury. There is a growing literature on the role of electrophysiological measures to guide prognosis, although the data is not consistent enough to allow robust guidance (51, 52). Generally, MMN and ERPs to own name stimuli are associated with a better prognosis, especially in those with MCS (53–57). Given that most patients have suffered from a multifocal brain injury to cortical and subcortical brain regions, one might expect systematic differences in the temporal and topographical features of their ERPs.

The MMN responses we observed were delayed by approximately a factor of two when compared with responses in healthy people (350–400 ms time window as opposed to 160–220 ms). Later components of the MMN have been reported in patients with DOC (53, 58, 59). Topographically, the right frontal region is often associated with a typical MMN response, where it has been interpreted as being driven by attentional rather than acoustical neural processing (60–62). By contrast, our posteriorly placed activation in sensor space is further back than late MMN components that have been reported in patients with MCS (53, 54).

The responsive groups SON ERPs were mainly located in left hemisphere consistent with other studies where the emphasis is on the linguistic/semantic aspects of the stimulus (63) rather than the relationship of the speaker to the listener, which tends to elicit a rightward response (64). Our main ERP was rather early, in the MMN range, which is compatible with our experimental design (comparing subject's own name to others' names) but the signage of the ERP was positive. As this comparison was within-subject and across time, it could represent a relative increase in one of the earlier, positive ERPs associated with hearing one's own name, such as the early nP3 rather than a change in an MMN-like response (65).

The results of the MMN and SON event related potential studies confirmed that, in the patients whose clinical scores improved, we also saw a commensurate improvement in objective measures of their cortical function. Future studies with larger patient numbers could allow to test these effects more robustly by comparing ERP changes in responders and non-responders. Although clinical assessment tools such as CRS-R are still the gold standard for diagnosis, neurophysiological tests such as ERPs can help to track responses to any given treatment more objectively and allow more reliable quantification of changes.

The most important outcome of this study was that these biophysical changes were accompanied by a statistically significant increase of CRS-R scores however, the absolute change was 1.4 points on the CRS-R scale with a mean value of 11.8. It is difficult to interpret this finding in a group of patients with a diagnosis of a permanent disorder of consciousness. A number of observations are worth making. First, it has to be recognized that DOC patients do vary in performance depending on time of day although reported variation is less than that observed in this study (66). It is possible that this mean change of 1.4 points reflected normal variation. Second, as this was an unblinded study it is possible that the finding of an increase of 1.4 reflects the observer bias. More positively, these results may reflect real clinical change. This view would be supported by the change in circadian rhythmicity, and sleep patterns which provide a mechanism for change and the electrophysiological improvement in cortical function which was associated with improvements in CRS-R score.

Our study findings are limited due to the small sample size and lack of control group. However, the 10 patients who were included in the interventional study had a definite diagnosis of their consciousness levels using SMART assessment in the first year of their brain injury and were monitored by experienced clinicians throughout their stay in the long term care unit. We were not able to study a control group as it was not possible to find another group of patients with matching etiology, age, consciousness levels and clinical features. Therefore, we decided to complete two baseline assessments to examine if the patients were showing signs of any spontaneous recovery, which is extremely rare after 2–8 years from brain injury or, showing any significant fluctuation of their consciousness levels.

A CRS-R score of 12 and above is associated with a good prognosis (67). As our knowledge of these conditions grows there is an understanding that a diagnosis of permanent disorder of consciousness is associated with a stable clinical trajectory (52). If this intervention has potential to change that trajectory it must be investigated carefully, as even small increases in awareness may allow patients to benefit further from stimulation programmes that take place in rehabilitation and care settings (68).

This small cohort study suggests that a simple, low cost intervention may have potential to improve outcome in patients with a disorder of consciousness. However, due to the small sample size our study should be considered as a preliminary study. The findings need to be evaluated further in larger cohorts in other centers, using other approaches to normalize circadian rhythm (such as ensuring feeding takes place during the day). While the intervention may be more difficult to implement in acute hospital settings which have 24 h activity, some consideration should be given to re-establishing circadian rhythm as early as possible. In addition, the studies should be extended to less severely brain injured patients where the circadian rhythm is also known to be disrupted and where normalizing it may improve outcome.

Clinically, this may be regarded as a low cost intervention which is easy to implement in rehabilitation and long term care settings and, carries a potential for positive impact on clinical outcomes.

## Data availability statement

The raw data supporting the conclusions of this article will be made available by the authors, without undue reservation.

## Ethics statement

Ethics approval was obtained from London Queen Square Research Ethics Committee (Ref: 11/LO/1233), patients' relatives were provided with a study information sheet, acted as consultees and signed a record of consultation form prior to the patient's enrolment in the study.

## Author contributions

KY is the main investigator for this study. AK, KY, and LJ contributed for data collection. SD, AL, SF, KY, and EP contributed for study design, data analysis, and publication. All authors contributed to the article and approved the submitted version.

## Funding

KY received research funding from the Royal Hospital for Neuro-disability. SF acknowledges personal funding support from the UCLH Biomedical Research Center.

## Conflict of interest

The authors declare that the research was conducted in the absence of any commercial or financial relationships that could be construed as a potential conflict of interest.

## Publisher's note

All claims expressed in this article are solely those of the authors and do not necessarily represent those of their affiliated organizations, or those of the publisher, the editors and the reviewers. Any product that may be evaluated in this article, or claim that may be made by its manufacturer, is not guaranteed or endorsed by the publisher.
