# Supplementary material for: A simple intervention for disorders of consciousness- is there a light at the end of the tunnel?
Source: Front Neurol. 2022 Jul 22;13:824880. doi: 10.3389/fneur.2022.824880 (PMC9355643; doi:10.3389/fneur.2022.824880)
Supplement: Supplementary file 1 [file Data_Sheet_1.PDF]

Table 1: CRS-R subscale scores for the patients who were included in the interventional study.

Pt ID	Auditory		Visual		Motor		Oromotor/ verbal		Communication		Arousal	
	Pre	Post	Pre	Post	Pre	Post	Pre	Post	Pre	Post	Pre	Post
Pt- 1	3	3	2	2	3	4	1	2	1	1	2	3
Pt- 2	2	2	2	2	2	3	1	1	0	0	2	2
Pt- 3	1	1	0	0	1	1	1	1	0	0	1	1
Pt- 4	1	1	3	3	2	2	1	1	0	0	2	3
Pt- 5	3	4	2	3	3	5	2	2	1	1	2	3
Pt-6	3	3	4	5	4	4	2	1	0	0	3	3
Pt-7	3	3	2	3	4	4	1	2	0	0	2	3
Pt- 8	2	3	3	3	1	3	2	2	0	0	3	2
Pt- 9	1	1	1	1	1	1	1	1	0	0	2	2
Pt-10	3	3	0	0	2	4	2	2	0	0	3	3

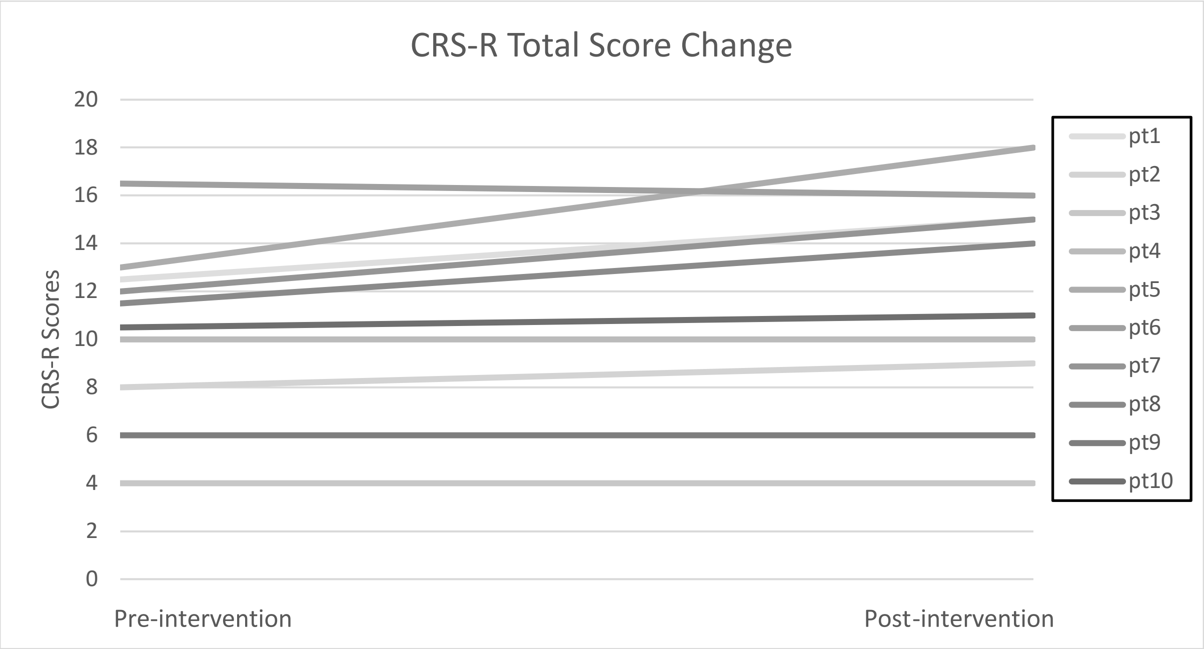

Figure 1: CRS-R total score change for the patients who were included in the interventional study.
